# Precision Targeting of Cardiac Macrophage Subsets to Ameliorate Heart Failure

**DOI:** 10.1016/j.jacbts.2024.10.013

**Published:** 2025-01-27

**Authors:** Connor Lantz, Edward B. Thorp

**Affiliations:** a Comprehensive Transplant Center, Department of Surgery, Northwestern University, Chicago, Illinois, USA; b Department of Pathology, Feinberg School of Medicine, Northwestern University, Chicago, Illinois, USA

**Keywords:** heart failure, cardiomyopathies

As the astronomer Carl Sagan wrote, “Biology is more like history than it is like physics. You have to know the past to understand the present.”^1^ This perspective can be applied to grasp the complexity of macrophages and their functions during both homeostasis and disease. For example, macrophages were once thought to be derived solely from monocytes; however, advanced genetic models now reveal that tissue macrophages originate from diverse lineages. These distinct developmental origins shape macrophage function within specialized tissues, allowing them to adapt uniquely to the local environment. Understanding macrophage origin can predict roles in immune responses, tissue repair, and the pathogenesis of various diseases.

During fetal development, yolk sac progenitors and fetal monocytes seed tissues to populate local resident macrophages, including the heart, while bone marrow–derived monocytes contribute to macrophage populations after birth.^2^^,^^3^ Once these progenitors arrive at their tissue of residence, they differentiate to fulfill roles within the tissue-specific niche. Despite organ-specific functional differences, core gene signatures are conserved across macrophages from different lineages and tissues. Among these, TLF^+^ macrophages, which express the markers TIM-4 (T cell membrane protein 4), LYVE-1 (lymphatic vessel endothelial hyaluronan receptor 1), or FOLR2 (folate receptor 2), are the most transcriptionally conserved population.^4^ These macrophages are primarily maintained through self-renewal, requiring minimal input from circulating monocytes. In contrast, CCR2^+^ macrophages are almost entirely dependent on monocyte replenishment.^4^ MHC-II^hi^ (TLF^‒^ CCR2^‒^) macrophages fall somewhere in between, as they are moderately replaced by monocytes but can also sustain themselves through a degree of self-renewal.^4^ This spectrum of monocyte dependency highlights the complexity of macrophage maintenance across different tissues and ages.

Within the heart, resident macrophages withstand the mechanical strain of the beating heart and participate in preserving cardiac homeostasis by phagocytosing defective mitochondria shed by cardiomyocytes, supporting cardiac energy demands.^5^ These resident cardiac macrophages arise from at least 3 distinct origins that are distinguished by the expression of TIM-4, CCR2 (C-C chemokine receptor 2), and MHC-II (major histocompatibility complex II).^6^^,^^7^ The unique response of each macrophage population to hypertensive stress underscores the importance of macrophage ontogeny. For example, in a model of angiotensin II–induced hypertension, TLF^+^ MHC-II^lo^ macrophages expand and secrete IGF-1 (insulin-like growth factor 1), promoting adaptive cardiomyocyte growth to help the myocardium endure hypertensive stress.^7^ Although TLF^+^ MHC-II^hi^ macrophages also proliferated, their overall numbers decreased during hypertension, and their contributions in this context remain underexplored.^7^ Therefore, further studies were necessary to elucidate how each cardiac macrophage population individually responds to pathological stressors and potentially contributes to the progression of cardiac disease.

In this issue of *JACC: Basic to Translational Science*, Zhang et al^8^ investigate the roles of TLF^+^ MHC-II^hi^ cardiac macrophages (referred to as double positive [DP]) during a pressure overload model of cardiac hypertrophy. The study demonstrates that DP macrophages first emerge in mice 42 days after birth, maintain stable populations throughout midlife, and decline later in life. Notably, DP macrophages are not limited to the heart, as they also reside in skeletal muscle and adipose tissue, but DP macrophages are either absent or present at very low levels in organs such as the kidney, liver, lung, and skin. Using advanced fate-mapping techniques, the investigators reveal that DP macrophages are maintained with minimal input from blood monocytes. Instead, these macrophages exhibit a moderate ability to proliferate, resembling self-renewing TIMD4^hi^ macrophages. However, MHC-II^hi^ and double-negative macrophages, are maintained through monocyte input. The authors’ findings align with previous studies on hypertension,^7^ further characterizing the self-renewing capacity of DP macrophages in the heart.

The investigators also examined how each resident cardiac macrophage population responds to transverse aortic constriction (TAC), a well-established model of pressure overload–induced cardiac hypertrophy and heart failure. Following TAC, the heart initially undergoes compensated hypertrophy corresponding to an acute decrease in the number of DP macrophages. While the DP macrophage population gradually rebounds after the acute phase, it significantly declines again during the late, decompensated phase of heart failure. In contrast, the number of TIMD4^hi^ macrophages slightly increases throughout both phases, likely due to enhanced proliferative activity. However, these findings rely on the assumption that each macrophage subset represents a discrete state, thus not accounting for the possibility that DP macrophages may down-regulate the surface expression of either TIMD-4 (phosphatidylserine receptor T cell immunoglobulin and mucin domain containing 4) or MHC-II in response to TAC. Despite this limitation, the data clearly indicate a decline in the DP macrophage population during both the acute phase of compensated hypertrophy and the late decompensated phase of heart failure.

Given the heterogeneity and diverse roles of cardiac macrophages, which can be beneficial or pathogenic, it is important to develop strategies that precisely target specific macrophage populations to better understand their role during cardiac pathologies. Previous approaches have focused on blocking monocyte recruitment to the myocardium or depleting all resident macrophages based on CX3CR1 expression. In this study, the authors utilized an innovative orthogonal recombinase system to selectively target the DP macrophage population. This approach employed adeno-associated virus (AAV) vectors encoding Cre under the LYVE-1 promoter and Dre under the MHC-II promoter, leading to the expression of a fluorescent marker and the diphtheria toxin receptor when both enzymes were present. Approximately 50% of DP macrophages were successfully labeled, and administration of diphtheria toxin resulted in a 33% reduction in the DP macrophage population. Although the deletion efficiency is modest, this reduction correlated with a decline in ejection fraction and increased myocardial fibrosis 4 weeks post-TAC. However, it remains unclear whether this strategy also inadvertently affects the TIMD4^+^ or MHC-II^hi^ populations, leaving room for further investigation into the specificity of this approach for resident macrophage depletion.

Single-cell RNA sequencing revealed that DP macrophages are highly enriched for *Retnla*, a gene that encodes resistin-like molecule alpha (RELMα). During myocardial infarction^6^ and sepsis-induced cardiomyopathy,^8^^,^^9^ RETNLA+ macrophages undergo self-renewal. Notably, precise targeting of DP macrophages in the context of TAC significantly decreased RELMα expression. To test if RELMα was sufficient to protect against TAC-induced injury, the investigators discovered that administration of RELMα promoted angiogenesis and decreased fibrotic deposition after TAC, resulting in an overall improvement of left ventricular function. Additionally, levels of resistin were elevated in patients with acute myocardial infarction or aortic stenosis, and higher resistin concentrations correlated with improved cardiac outcome markers in these patients. These findings suggest that resistin could serve as a potential clinical biomarker for predicting prognosis in heart failure patients.

In summary, the innovative approaches utilized by Zhang et al^8^ shed light on the complex dynamics of DP macrophages, revealing their distinct response to pressure overload cardiac hypertrophy and heart failure. By elucidating the origins, maintenance, and functional capacities of separate macrophage populations, the authors’ research highlights the need for targeted strategies that can manipulate specific subsets to enhance cardiac resilience or mitigate damage. Moreover, the identification of RELMα as a key factor for cardioprotection offers promising avenues for therapeutic intervention and the potential for using resistin as a biomarker for heart failure prognosis. Continued investigation into the specific contributions of various macrophage populations will be informative in supplementing effective therapies aimed at improving cardiac outcomes and understanding the underlying mechanisms of heart disease and inflammation.

## Funding Support and Author Disclosures

The authors have reported that they have no relationships relevant to the contents of this paper to disclose.

## References

[1] Goodreads Carl Sagan quotes. https://www.goodreads.com/quotes/9178812-biology-is-more-like-history-than-it-is-like-physics.

[2] Hashimoto D., Chow A., Noizat C. (2013). Tissue-resident macrophages self-maintain locally throughout adult life with minimal contribution from circulating monocytes. Immunity.

[3] Schulz C., Perdiguero E.G., Chorro L. (2012). A lineage of myeloid cells independent of Myb and hematopoietic stem cells. Science.

[4] Dick S.A., Wong A., Hamidzada H. (2022). Three tissue resident macrophage subsets coexist across organs with conserved origins and life cycles. Sci Immunol.

[5] Nicolás-Ávila J.A., Lechuga-Vieco A.V., Esteban-Martínez L. (2020). A network of macrophages supports mitochondrial homeostasis in the heart. Cell.

[6] Dick S.A., Macklin J.A., Nejat S. (2019). Self-renewing resident cardiac macrophages limit adverse remodeling following myocardial infarction. Nat Immunol.

[7] Zaman R., Hamidzada H., Kantores C. (2021). Selective loss of resident macrophage-derived insulin-like growth factor-1 abolishes adaptive cardiac growth to stress. Immunity.

[8] Zhang D., Wang X., Zhu L. (2025). TIMD4^hi^MHCII^hi^ macrophages preserve heart function through Retnla. JACC Basic Transl Sci.

[9] Zhang K., Wang Y., Chen S. (2023). TREM2hi resident macrophages protect the septic heart by maintaining cardiomyocyte homeostasis. Nat Metab.

